# Standard Total Ankle Arthroplasty vs. Patient-Specific Instrumentation: A Comparative Study

**DOI:** 10.3390/jpm14070770

**Published:** 2024-07-19

**Authors:** Alberto Arceri, Pejman Abdi, Antonio Mazzotti, Simone Ottavio Zielli, Elena Artioli, Laura Langone, Federico Sgubbi, Cesare Faldini

**Affiliations:** 1 1st Orthopaedics and Traumatologic Clinic, Istituto di Ricovero e Cura a Carattere Scientifico (IRCCS), Istituto Ortopedico Rizzoli, 40136 Bologna, Italy; alberto.arceri@ior.it (A.A.); pejman.abdi@ior.it (P.A.); simoneottavio.zielli@ior.it (S.O.Z.); elena.artioli@ior.it (E.A.); laura.langone@ior.it (L.L.); federico.sgubbi@ior.it (F.S.); cesare.faldini@ior.it (C.F.); 2 Department of Biomedical and Neuromotor Sciences (DIBINEM), Alma Mater Studiorum University of Bologna, 40123 Bologna, Italy

**Keywords:** total ankle arthroplasty, patient-specific instrumentation, surgical outcomes, retrospective analysis, complications

## Abstract

Purpose: This retrospective study aims to compare surgical outcomes between two cohorts of patients who underwent total ankle arthroplasty (TAA) using either standard technique or patient-specific instrumentation (PSI). Methods: A consecutive series of patients who affected of end-staged ankle osteoarthritis were retrospectively assessed and divided into two groups based on TAA techniques: a TAA standard technique group and a TAA-using PSI group. The two groups were compared in terms of operative time, additional procedures, complications (neurovascular and wound problems, infection, loosening and osteolysis, revision and explantation rates, and perioperative fracture), clinical scores, and range of motion (ROM). Result: Fifty-one patients underwent standard TAA, while 13 patients underwent TAA with PSI. At 1-year follow-up, there were no significant differences in complication rates between the two groups (*p* > 0.05). AOFAS scores were similar, with the standard TAA group scoring 83.33 ± 7.55 and the PSI group scoring 82.92 ± 9.7 (*p* = 0.870). Likewise, the postoperative ROM did not differ significantly, with 15.12 ± 7.6 degrees for the standard TAA group and 16.05 ± 6.7 degrees for the PSI group (*p* = 0.689). However, the standard TAA group experienced significantly longer operative time (107.1 ± 22.1 min) compared to the PSI group (91.92 ± 22.9 min, *p* = 0.032). Additionally, the standard TAA group required more adjunctive procedures (29.7%) compared to the PSI group (7.7%, *p* = 0.04). Residual pain was also more frequently reported in the standard TAA group (62.7%) than in the PSI group (30.7%, *p* = 0.038). Conclusion: While both techniques resulted in comparable complication rates, clinical scores and ROM, the PSI group reported significantly shorter operative time and less residual pain, thus requiring fewer postoperative procedures.

## 1. Introduction

Total ankle arthroplasty (TAA) is an effective and increasingly viable surgical procedure for end-stage ankle osteoarthritis with an overlapping functional outcome [1,2,3]. In contrast to ankle fusion, TAA aims to restore motion and requires the restoration of joint anatomy and biomechanics. This speculation undoubtedly needs precision in bone cutting and prosthetic components positioning. To enhance these aspects, there have been numerous advancements in implant design and surgical techniques in recent years. One notable innovation is the introduction of patient-specific instrumentation (PSI) in TAA. This is a custom-made surgical device based on preoperative computed tomography (CT) scans and computer-generated three-dimensional (3D) models. PSI has the theoretical potential to improve accuracy in bone resection, implant positioning, and alignment and thus should result in satisfactory outcomes [4,5]. The potential advantages of using PSI also include a reduction in procedural complexity and a decrease in operative time [5], which could potentially lead to a lower incidence of complications.

Current literature is focused on the accuracy propriety of a PSI in TAA, demonstrating that a PSI system accurately and reliably assists in total ankle prosthesis implant positioning, except for the prediction of implant size [6,7]. However, limited comparative studies exist evaluating the efficacy of PSI versus standard techniques in TAA regarding operative time, additional procedures, complications (neurovascular and wound problems, infection, loosening and osteolysis, revision and explantation rates, and perioperative fracture), clinical scores, and range of motion (ROM). By examining these variables, it may be possible to better understand whether the theoretical advantages of PSI manifest in practical clinical benefits, thus offering valuable insights for orthopedic surgeons considering its adoption in TAA procedures.

Our hypothesis is that the enhanced precision of PSI, as demonstrated in various studies, should translate into improved intraoperative and postoperative clinical outcomes when compared to standard TAA techniques. We anticipate that PSI will reduce operative time, require fewer adjunctive procedures, and be associated with fewer complications while yielding superior clinical scores and ROM.

Therefore, the purpose of this study is to comprehensively compare the surgical outcomes between standard TAA and TAA performed using PSI.

## 2. Materials and Methods

### 2.1. Patient Selection

A retrospective study, using our institutional database of all patients who underwent standard TAA or TAA with PSI from January 2020 to December 2022, was conducted.

The protocol was approved by the ethical committee and all patients provided informed consent. The study was conducted in accordance with the ethical principles outlined in the Declaration of Helsinki and with the Guidelines for Good Clinical Practice. The study followed the STROBE statement and checklist for retrospective studies [8].

Specific inclusion criteria were used to select participants for our study to ensure the relevance and reliability of our findings. (1) Patients diagnosed with end-stage ankle osteoarthritis who underwent TAA were included in the study. Specifically, this included those who received either the standard TAA technique or TAA with PSI. (2) To ensure that differences in outcomes were more accurately attributed to the type of instrumentation used (standard vs. PSI) rather than to differences in surgical approach, we only included those who received either technique via an anterior surgical approach, which is routinely used at our institute. (3) Only patients who had a complete set of pre- and postoperative clinical and radiographic evaluations with a minimum follow-up of 1 year were included in the study.

Exclusion criteria were (1) revision arthroplasty or severe bone loss, (2) patients with previous ankle fusions, and (3) ankle with deformity in varus/valgus over 10 degrees and intra-articular incongruency. The 10-degree cut-off was established based on prior studies, which suggest that a deformity exceeding 10 degrees serves as an indication to perform additional bony procedures, such as osteotomies and/or adjacent joint fusions [9,10,11]. Furthermore, if the tibiotalar joint is incongruent, certain studies advocate balancing by medial/lateral malleolar lengthening osteotomy, lateral or medial ligament repair/reconstruction, or release, whereas if there is intra-articular congruency, ligament-balancing TAA may be feasible, usually obviating the need for additional local surgery [12,13,14].

These criteria were meticulously designed to create a homogeneous patient population that would allow for a robust and meaningful comparison between standard TAA and TAA with PSI.

### 2.2. Surgical Techniques

Two experienced foot and ankle orthopedic surgeons were responsible for performing the TAAs. One surgeon exclusively performed the TAAs using the standard technique, while the other focused exclusively on the PSI approach. Due to the nature of the procedures, the surgeons were necessarily aware of the technique they were using. However, efforts were made to ensure blinding in the evaluation process. Independent clinicians, specifically radiologists for radiographic assessments and orthopedic residents who administered clinical scores to the patients, conducted these assessments without knowledge of the surgical technique used. This approach was designed to facilitate an unbiased comparison between the outcomes of the two different surgical methods.

The standard TAA employed the FAR^®^ mobile-bearing prosthesis (AdlerOrtho, Milan, Italy) was executed via an anterior approach. The talar and tibial resections were conducted using a conventional tibial alignment jig and cutting blocks, followed by the implantation of cementless talar and tibial components, along with an ultra-high molecular weight polyethylene (UHMWPE) meniscus.

**Figure 1:**
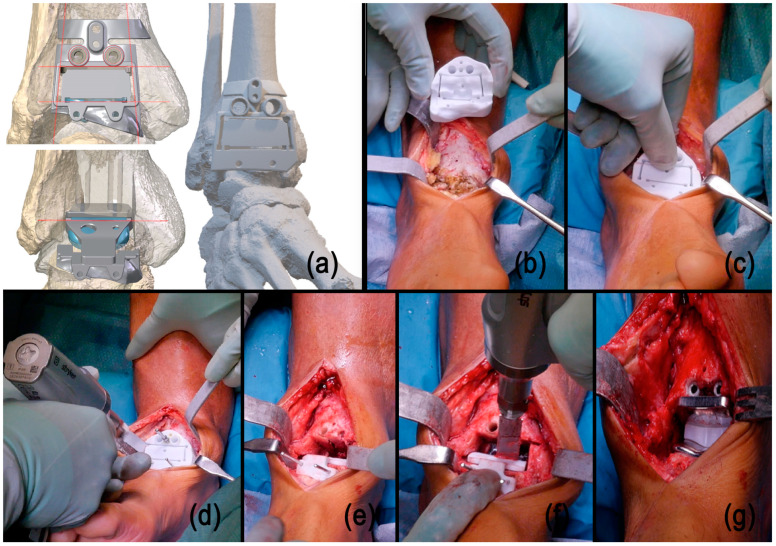
Preoperative CT scans were used to create a three-dimensional model of the ankle, which was used to customize cutting guides (**a**). The first cutting guide was precisely designed and manufactured to align with the anterior aspect of the joint, including the osteophytes (**b**,**c**). Using an oscillating bone saw, talar and tibial resections were carried out through the first cutting guide, which was stabilized with Kirschner wires (**d**). The second talar cutting guide was then positioned and stabilized using K-wires. This guide facilitated the posterior chamfer resection and included two holes for the preparation of the talar bone pegs (**e**,**f**). The final implant was correctly placed into the prepared bone resections (**g**).

For TAAs utilizing PSI, preoperative CT scans were used to generate a three-dimensional model of the ankle. This model facilitated the customization of cutting guides for the ankle. A virtual implantation was conducted to ensure precise component positioning, and the patient-specific cutting guides were then fabricated based on this simulation. The surgical procedure was performed using these guides through an anterior approach, preserving necessary osteophytes to maintain tactile feedback. Specifically, the distal tibial and talar resections were performed using the patient-specific guides [15,16] (Figure 1).

In both the standard and PSI procedures, the ankle was immobilized in a plaster cast for three weeks, during which weight-bearing was prohibited. Following cast removal, progressive weight-bearing was permitted with the use of a walking boot, and flexion–extension exercises for the ankle were encouraged [17].

### 2.3. Outcomes Assessment

Patient and operative data were collected by manual chart review. Patient information included age and gender, while operative data included operative time, any subsequent postoperative additional bony procedures, revisions or explantations. Postoperative additional bony procedures include any adjunctive surgical procedures, as supramalleolar or calcaneal osteotomy, performed to correct residual deformities after TAA.

Complications, including the occurrence of neurovascular and wound problems, infection, loosening and osteolysis, revision and explantation rates, and perioperative fracture, were assessed at the clinical and radiological follow-up visits.

Residual pain was routinely assessed during the hospitalization and at follow-up visits using the Numerical Rating Scale (NRS), wherein pain intensity during daily activity is rated from 0–10, where 0 indicates no pain and 10 indicates the worst pain [18]. NRS at 1-year follow-up was collected as reference of comparison.

All patients were administered the American Orthopedic Foot and Ankle Society Ankle–Hindfoot (AOFAS-AH) Scale questionnaires both pre- and postoperatively [19]. Clinical outcomes were graded for pain (from 0 to 40 points), functional evaluation (from 0 to 50 points), and alignment (from 0 to 10 points). Thus, the overall score can range from 0 to 100 points, where 100 is a foot without pain, plantigrade, ankle–hindfoot well aligned, and no functional or gait issues. Preoperative scores were compared with ones collected at 1 year follow-up visits.

ROM was measured radiographically using an established method on postoperative weight-bearing lateral ankle radiographs, including maximal dorsiflexion, maximal plantarflexion, and total ROM [20].

### 2.4. Statistical Analysis

Data collection was carried out utilizing Microsoft Excel Version 365 (Microsoft Corporation, Redmond, Washington, DC, USA) for Windows 11, and statistical analysis was performed using the software Jamovi project (2022) version 2.3.

The primary parameters considered for the power analysis to determine the appropriate sample size were operative time and ROM. The alpha level was set at 0.05, with a study power of 80%. To estimate the effect size for operative time, the study by Saito et al. [21] was referenced, which reported an average operative time of 167 ± 42 min in the PSI group and 190 ± 46 min in the standard group. For the ROM effect size, data from study by Marks et al. [22] were utilized, which provided a mean postoperative ROM for standard TAA of 40.0 ± 12.3°. In the absence of a direct comparison for the PSI group, the significant average improvement in tibiotalar total ROM of 8.1° reported by Fletcher et al. [23] was used.

Continuous variables were reported as mean and standard deviation, while categorical variables were reported as percentage and frequency. Variables were analyzed for normality using the Shapiro Wilk test. Statistical analysis was performed using chi-square testing when appropriate to determine differences in categorical variables between groups, while differences in continuous variables were analyzed using Student’s *t*-test.

Statistical significance was set at *p* value less than 0.05 per standard convention.

## 3. Results

### 3.1. Population

**Figure 2:**
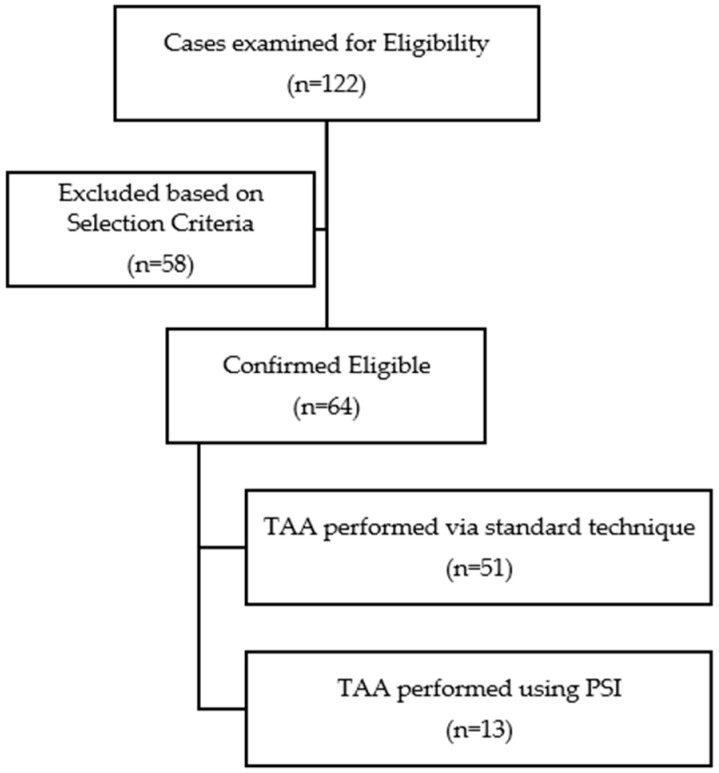
Flowchart of the study cohort.

From a chart review, 122 patients who had undergone TAA were identified. Out of these, 64 patients fulfilled the selection criteria and were included in the study (Figure 2).

The study involved 64 patients who underwent TAA. Out of these, 51 patients received standard TAA, while 13 patients underwent TAA with PSI. Their characteristics are shown in Table 1. No statistical differences were found between the two groups. The mean follow-up was 18.6 months.

### 3.2. TAA vs. TAA Using PSI

**Figure 3:**
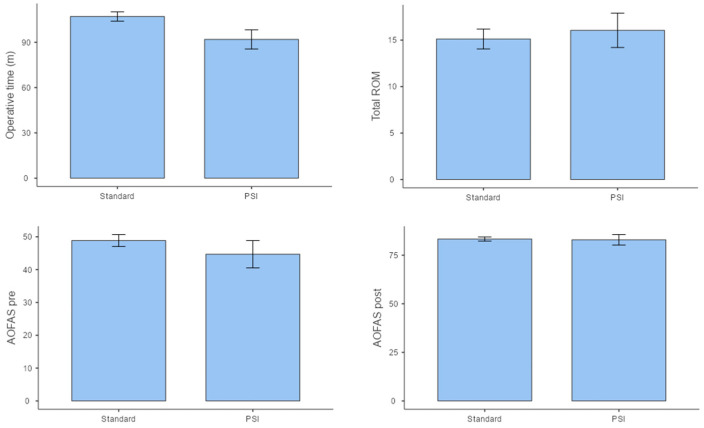
A bar graph showing the mean of the main outcomes for both groups, with standard error bars.

Comparison between the standard TAA and PSI groups revealed no statistically significant difference in complications rate, but only in operative time (Table 2). However, the standard TAA group showed a significantly higher frequency of additional procedures (*p* < 0.05) (Table 3 and Figure 3). Regarding clinical outcomes, the TAA with PSI group reported less residual pain compared to the standard TAA group (*p* < 0.05). No statistical differences in AOFAS score and ROM were found between the two groups (Table 3 and Figure 3).

Given our current sample sizes, the power analysis suggests that our study may be underpowered for detecting differences in outcomes, which may require cautious interpretation.

## 4. Discussion

The findings of this study suggest comparable overall outcomes between standard TAA and PSI in terms of operative time, complication rate, and clinical outcomes. However, the standard TAA group reported longer operative time and higher residual pain and required more postoperative surgical procedures compared to PSI group.

Significant reductions in operative time, tourniquet time, and radiation exposure in the PSI group were already reported in the majority of studies [4,5,21,24,25,26,27]. Our findings corroborate this significant difference (Table 2). However, it is important to note that this advantage may not always be as pronounced. Operative time is influenced by the surgeon’s skill. Specifically, inexperienced surgeons may benefit more from PSI due to its tailored guidance, whereas experienced surgeons who are proficient in standard TAA techniques might achieve similar operative time without PSI.

To our knowledge, no studies have specifically investigated whether PSI reduces the need for concomitant or staged additional procedures to TAA. Theoretically, the use of software to plan tibial and talar cuts could improve the correction of ankle and hindfoot alignment in frontal plane deformities, when the deformity is less than 10 degrees and the tibiotalar joint remains congruent (thus not requiring additional ligamentous procedures). Implanting a prosthesis with cuts parallel to the existing deformity may leave a residual inframalleolar deformity. Attempting to correct the deformity intraoperatively with a standard TAA is challenging due to the loss of anatomical landmarks and the non-weight bearing status of the ankle, which may result in suboptimal postoperative alignment at follow-up.

Our case series demonstrated a significant reduction in the need for adjunctive procedures in the PSI group (Table 2). Therefore, PSI could play a critical role in the management of ankle deformities < 10 degrees in the frontal plane with a congruent tibiotalar joint. This approach may reduce the need for concurrent or staged corrective procedures after TAA.

The existing literature regarding complications associated with PSI TAA is limited. The studies that do report complications suggest that there is no significant difference in revision and complication rates between PSI and standard TAA [7,26,28]. However, standard TAA group reported higher residual pain compared to PSI group (Table 2). The observed results may be attributed to the precision of PSI in positioning and orientation the prosthetic components in the axial plane, which could potentially prevent component malrotation and reduce the risk of gutter impingement, a common source of residual pain post-TAA [29]. Nonetheless, it should be noted that some degree of residual pain has been reported even in the PSI group (Table 2).

In general, previous papers showed significant differences in favor of PSI on the walking/standing domain of the MOXFQ [27] and the total AOFAS score [26] at 12 months; however, in our case series, no differences in AOFAS score were found between the two groups (Table 3).

To date, comparative studies regarding ROM between standard TAA and PSI TAA have been lacking. This study represents the first attempt to compare ROM between these two groups, but no statistical differences in ROM was found (Table 3). The ROM values observed in our case series are consistent with those reported in the current literature [22,30,31]. However, when considering the prosthetic model most similar to the one used in our case series, the mean values for plantar flexion are lower than those reported in the literature [32].

Although the current results do not allow for definitive conclusions, it is crucial to recognize the important clinical implications of this type of study. If PSI demonstrates a reduction in operative time and complication rates, it could become the preferred method for performing TAA, especially in more complex cases or when performed by less experienced surgeons. Furthermore, should the investigation show that PSI leads to superior clinical scores and improved ROM, PSI could be established as a more effective technique for restoring ankle function and mobility compared to the standard approach. Overall, these findings could significantly influence surgical practices and decision-making processes, potentially leading to the broader adoption of PSI in ankle replacement surgeries and setting new benchmarks in the field.

The limitations of this study include its retrospective design and the small size of the sample, particularly for the PSI group. The limited sample size in our study is primarily attributable to the relatively low volume of this surgery and the stringent selection criteria employed. TAA and especially the PSI application are specialized procedures that are not performed in high frequencies at most medical centers. Moreover, our study applied rigorous inclusion and exclusion criteria to minimize confounding variables and enhance the reliability of our findings. The smaller sample size reflects both the rarity of the procedure and our commitment to maintaining high methodological standards. Despite the limited number of participants, our study offers valuable comparative insights into the standard TAA and PSI techniques. However, we acknowledge the potential limitations in statistical power. These factors underscore the need for continued investigation and larger-scale studies to fully elucidate the comparative benefits and risks of these approaches in TAA. To address these challenges, future studies should consider strategies such as multi-center collaboration to increase the sample size, the collection of more detailed baseline data to improve effect size estimation, and potentially, the use of advanced statistical methods that can handle small sample sizes and high variability. Additionally, ongoing data collection and analysis will help refine effect size estimates, thereby facilitating more accurate sample size calculations in subsequent research.

An additional limitation of our study is the absence of a preoperative assessment of ROM, which could be a crucial parameter that can significantly influence postoperative outcomes. Future studies should include this measure to provide a more comprehensive analysis.

Finally, to better evaluate the long-term clinical and functional outcomes, and to determine the sustained effectiveness of PSI in TAA, a follow-up period extending beyond one year is essential.

Future studies could explore computational simulations or in silico modeling [33,34] to further investigate this topic. Computational simulations could provide valuable insights into biomechanical aspects such as joint kinematics, stress distribution on implants, and functional outcomes. By simulating different surgical scenarios and patient-specific anatomies, researchers can potentially predict the impact of PSI on surgical precision, implant positioning, and overall ankle joint function. Such studies could complement clinical findings, offering a deeper understanding of how PSI influences surgical outcomes and guiding refinements in surgical techniques and implant designs.

## 5. Conclusions

While both techniques resulted in comparable complication rates, the PSI group reported significantly shorter operative time and less residual pain, thus requiring fewer postoperative procedures. Although the current results do not allow definitive conclusions to be drawn, these preliminary findings suggest that the PSI technique may offer potential advantages due to the improved reliability and accuracy of prosthetic implant positioning provided by the guided approach of PSI. This may be particularly beneficial in more complex cases or when performed by less experienced surgeons.

Further research efforts with larger sample sizes and rigorous study designs are warranted to provide clearer insights into the comparative efficacy between standard TAA and TAA with PSI.

## Figures and Tables

**Table 1:** Patient’s characteristics.

	Standard TAA	PSI TAA	*p*-Value
Patients	51	13	
Age	59.9 ± 10.3	55.6 ± 12.1	0.344
Gender	17 M/34 F	5 M/8 F	
OA etiology	2 PRIM	1 PRIM	
	1 RA	1 RA	
	1 C	11 PT	
	47 PT		
OA onset	5.6 ± 4.7	6.7 ± 4.5	0.543

Abbreviations: M male; F female; PRIM primitive; RA rheumatoid arthritis; C clubfoot; PT post-traumatic; OA osteoarthritis.

**Table 2:** Standard and PSI TAA comparison of operative time and complications.

	Standard TAA (51)	PSI (13)	*p*-Value	Cohen’s d	95% CI for Cohen’s d Lower Upper	
Operative time (min)	107.1 ± 22.1	91.92 ± 22.9	0.032 *	0.68	0.05	1.30
Additional procedure (%)	19 (29.7)	1 (7.7)	0.04 *			
Revision (%)	5 (9.8)	2 (15.3)	0.565			
Explantation (%)	3 (5.8)	1 (7.7)	0.81			
VNP complication (%)	2 (3.9)	2 (15.3)	0.127			
Wound problem (%)	10 (19.6)	4 (30.7)	0.385			
Infection (%)	3 (5.8)	1 (7.7)	0.81			
Fracture (%)	2 (3.9)	0 (0)	0.468			
Loosening (%)	5 (9.8)	2 (15.3)	0.565			
Residual pain (%)	32 (62.7)	4 (30.7)	0.038 *			

Abbreviations: TAA total ankle arthroplasty; PSI patient-specific instrumentation, VNP neurovascular, CI confidence intervals. * Indicates reaching statistical significance (*p* < 0.05).

**Table 3:** Standard and PSI TAA comparison of preoperative and postoperative AOFAS outcomes and postoperative ROM.

		Standard TAA (51)	PSI TAA (13)	*p*-Value	Cohen’s d	95% CI for Cohen’s d Lower Upper	
Preoperative	AOFAS tot	48.86 ± 12.92	44.69 ± 15.02	0.319	0.31	0.30	0.92
	Pain	10.98 ± 10.05	7.69 ± 10.13	0.297	0.33	−0.29	0.94
	Function	26.75 ± 9.44	25.77 ± 9.71	0.742	0.1	−0.50	0.71
	Alignment	11.14 ± 3.84	11.23 ± 3.63	0.937	−0.02	−0.63	0.58
Postoperative	AOFAS tot	83.33 ± 7.55	82.92 ± 9.7	0.87	0.51	−0.56	0.66
	Pain	24.90 ± 6.12	25.38 ± 6.6	0.804	−0.07	−0.69	0.53
	Function	46.1 ± 3.82	45.0 ± 4.53	0.376	0.27	−0.34	0.88
	Alignment	12.33 ± 3.37	12.54 ± 4.52	0.856	−0.05	−0.66	0.55
ROM postop	Total	15.12 ± 7.6	16.05 ± 6.7	0.689	−0.12	−0.73	0.48
	Dorsiflexion	6.03 ± 3.4	5.79 ± 3.5	0.822	0.07	−0.54	0.68
	Plantarflexion	9.07 ± 5.5	10.24 ± 4.4	0.48	−0.22	−0.83	0.39

Abbreviations: TAA total ankle arthroplasty; PSI patient-specific instrumentation, CI confidence intervals.

## Data Availability

Some or all data and models that support the findings of this study are available from the corresponding author upon reasonable request.
